# TGF-β Regulates m^6^A RNA Methylation after PM_2.5_ Exposure

**DOI:** 10.3390/toxics11121026

**Published:** 2023-12-16

**Authors:** Tingting Wu, Bingqian Liu, Yongjie Wei, Zhigang Li

**Affiliations:** State Key Laboratory of Environmental Criteria and Risk Assessment, Chinese Research Academy of Environmental Sciences, Beijing 100012, China; wutingting777@yeah.net (T.W.); liubingqian1118@yeah.net (B.L.); weiyj@craes.org.cn (Y.W.)

**Keywords:** PM_2.5_, m^6^A, RNA methyltransferase, TGF-β, A549

## Abstract

PM_2.5_ exposure leads to a variety of respiratory diseases, including pulmonary fibrosis, metastatic lung cancer, etc. Exposure to PM_2.5_ results in the alteration of epigenetic modification. M^6^A RNA methylation is an essential epigenetic modification that regulates gene expression at the post-transcriptional level. Our previous study found that PM_2.5_ exposure up-regulated m^6^A RNA methylation and TGF-β expression level in the lung, but the mechanisms and pathways of PM_2.5_ regulation of m^6^A RNA methylation are still unclear. Moreover, a previous study reported that the TGF-β signal pathway could regulate m^6^A RNA methylation. Based on this evidence, we investigate the role of the TGF-β signaling pathway in PM_2.5_-induced m^6^A RNA methylation with the A549 cell line. Our results showed that PM_2.5_ could induce upregulation of m^6^A RNA methylation, accompanied by increased expression of TGF-β, Smad3, methyltransferase-like 3 (METTL3), methyltransferase-like 14 (METTL14). Furthermore, these alterations induced by PM_2.5_ exposure could be reversed by treatment with TGF-β inhibitor. Therefore, we speculated that the TGF-β signal pathway plays an indispensable role in regulating m^6^A RNA methylation after PM_2.5_ exposure. Our study demonstrates that PM_2.5_ exposure influences m^6^A RNA methylation by inducing the alteration of the TGF-β signal pathway, which could be an essential mechanism for lung-related diseases induced by PM_2.5_ exposure.

## 1. Introduction

Atmospheric fine particulate matter with a size of less than 2.5 μm (PM_2.5_) plays a leading role in the adverse health effects [1]. Numerous epidemiological investigations have shown that PM_2.5_ exposure increases the risk of respiratory disease [2]. PM_2.5_ has a small diameter, which allows it to enter the lungs and be deposited through respiration. PM_2.5_ could enter the alveolar cells or bloodstream, worsen asthma and rhinitis, induce pulmonary fibrosis, and promote the migration and invasion of lung cancer cells [3,4]. Toxicological research has pointed out some reference pathogenesis of PM on cardiopulmonary effects, including inflammation, oxidative stress, and imbalance of the autonomic nervous system [5,6]. However, the specific molecular mechanisms and regulatory pathways of PM_2.5_-induced adverse health outcomes are still being explored.

Environmental changes and atmospheric pollution are the major contributors to epigenetic modification. In recent years, many studies have focused on an important form of epigenetic modification, RNA methylation, which regulates gene expression at the post-transcriptional level [7]. N^6^-methyladenosine (m^6^A), the methylation modification of the sixth N of adenine on RNA, accounts for the most significant proportion of RNA methylation [8]. M^6^A RNA methylation is adenylate undergoes methylation modification at the sixth N position by methylation enzymes, such as RNA methyltransferase-like 3 (METTL3), methyltransferase-like 14 (METTL14), and Wilms’ tumor 1-associating protein (WTAP) [9]; m^6^A RNA methylation can also be removed by RNA demethylases such as fat mass and obesity-associated protein (FTO), de-methyltransferase ALKB homolog 5 (ALKBH5) [10]. It has been well established that m^6^A RNA methylation is involved in cell apoptosis, cell proliferation, and differentiation, and many other life activities [11]. Our previous study showed that acute PM_2.5_ exposure induced an up-regulation of RNA m^6^A levels, and increased expression of the METTL3 and METTL14 in the lungs of mice [12]. Our previous study has found that acute PM_2.5_ exposure induced an up-regulation of RNA m^6^A levels and increased expression of the METTL3 and METTL14 in mice’s lungs [12]. Many research studies reported that related factors that regulate m^6^A could affect spermatogenesis (ALKBH5, METTL3), development (METTL3, FTO, ALKBH5), immunity (METTL3), UV-induced DNA damage response (METTL3, FTO), tumor metastasis (METTL14), stem cell renewal (METTL14), adipocyte differentiation (FTO), cellular development and differentiation, cell division, and several other life processes [13]. This evidence suggests that m^6^A modifications regulate RNA stability, localization, transport, shearing, and translation at the post-transcriptional level and perform critical biological functions in mammals [14,15].

However, the mechanisms and pathways of PM_2.5_ regulation of m^6^A RNA methylation are still unclear. Some toxicological studies have pointed out that PM exposure can increase TGF-β expression [16,17]. It was demonstrated that activation of TGF-β and its receptors lead to phosphorylation of Smad protein, which is an essential pathogenic mechanism in pulmonary fibrosis [18]. Moreover, Bertero et al. [19] pointed out that activated phosphorylated Smad2/3 can enter the nucleus and bind to RNA methyltransferase complex, then induce m^6^A RNA methylation in pluripotent stem cells. Preliminary studies conducted in our team have indicated that exposure to atmospheric particulate matter can alter the overall levels of RNA m^6^A and TGF-β expression in the lungs. Given the above evidence, we hypothesize that PM_2.5_ exposure affecting downstream m^6^A RNA methylation levels through regulation of TGF-β could be a pathway to trigger adverse health effects. Therefore, this study is aimed to clarify the role of the TGF-β signal pathway in PM_2.5_-induced m^6^A RNA methylation. This study helps to clarify the molecular mechanism of PM_2.5_-induced adverse health endpoints and provides a scientific basis for further development of intervention measures.

## 2. Materials and Methods

### 2.1. Animals and Ethical Approval

Male C57BL/6 mice (6–8 weeks old) were purchased from the Charles River, Beijing, China. The mice were acclimatized in a host animal house facility for 14 days with pathogen-free cages at 24 ± 1 °C and 55–75% humidity with a 12 h light-dark cycle. Mice were provided with ad libitum commercially available diet and filtered water. This study was approved by the Scientific Research Ethics Committee, Chinese Research Academy of Environmental Sciences (Project reference No: 006–2019) Rules of the Committee for Control and Supervision of Experiments on Animals were followed for the study.

Our published article described the exposure protocol in detail [12]. Here we set the three groups of mice in the experiment: (a) the control group (Ctrl), in which mice were exposed in the chamber with high-efficiency particulate air filters (HEPA) for 24 h, had a mean PM_2.5_ concentration of 17.9 ± 7.8 μg/m^3^; (b) the PM_2.5_ group (PM_2.5_), in which mice were exposed to directly introduced ambient air in the chamber for 24 h, had a mean PM_2.5_ concentration of 271.6 ± 84.8 μg/m^3^; (c) the air purification group (Reversal), mice were transferred to the Ctrl chamber after 24 h of exposure in the PM_2.5_ chamber for another 120 h of continuous exposure, and the mean PM_2.5_ concentration was 12.4 ± 4.2 μg/m^3^.

### 2.2. PM_2.5_ Sampling and Suspension Preparation

PM_2.5_ was collected outdoors from November 2019 to February 2020. The average temperature and humidity during the sampling period are shown in Table 1. PM_2.5_ sampler (Dandong Better Instruments Co., Heilongjiang, China) was set at the Chinese Research Academy of Environmental Sciences, Beijing. The sampling flow rate was 16.67 L/min. PM_2.5_ was collected with Teflon membrane. Teflon membrane equilibrate in a constant temperature and humidity (20~25 °C, 50% humidity) box prior to this collection for 24 h, weigh the membrane with a 0.01 mg electronic scale (MS105 Mettler Toledo, Zurich, Switzerland) prior to sampling, weigh again after sampling, and record the difference in membrane weights. The collecting sampled membranes were stored at −20 °C until analysis. Each membrane is collected for 23 h (4:00 p.m. to 3:00 a.m. the next day).

The following is the process of PM_2.5_ suspension preparation. The Teflon membranes loaded with PM_2.5_ were ultrasonically extracted with ultrapure water. The mixture was then freeze-dried, and the solids were accurately weighed using a one ten-thousandth balance. Finally, the solids were mixed with DMEM medium without serum to form PM_2.5_ suspension. PM_2.5_ suspension was stored at 4 °C and protected from light before use.

### 2.3. Cell Culture and PM_2.5_ Exposure

The A549 cells were used in the present study. Due to its advantages of easy culture, it was widely used in many studies [20]. A549 is genotypically stable and has an extremely high differentiation state under different culture conditions in vitro [21]. The cells secrete a large number of factors and chemicals that are involved in a wide range of physiology and pathology. Therefore, A549 can be used as a model to research the toxicology of air pollutants. The A549 cell line was purchased from the Shanghai Institute of Cell Biology. A549 cells were grown in DMEM (Gibco BRL, Grand Island, NY, USA) containing 10% calf serum (N-10) in a 5% CO_2_ incubator at 37 °C. The cells were seeded in a six-well plate at 1 × 10^6^ cells per well, and then were exposed to PM_2.5_ suspension for 24 h. The final concentration gradient of PM_2.5_ treatment is preset to be 0, 50, 100, and 200 μg/mL. The cells were collected and frozen at −80 °C for analyses after PM_2.5_ exposure. All cell experiments were performed three times, and the results are presented as mean values ± standard deviation.

### 2.4. Cell Membrane Permeability Analysis

HCS LIVE/DEAD^®^ Green Kit (Catalog no. H10290, Invitrogen, Waltham, MA, USA) with a two-color nuclear fluorescence staining assay is used to evaluate the cell viability after PM_2.5_ exposure [22]. The kit includes Image-iT DEAD Green™ viability stain for discrimination of dead cells and HCS NuclearMask™ Deep Red stain for total cell demarcation. The Image-iT^®^ DEAD Green™ viability stain in the kit is impermeable to healthy cells, whereas it can enter once the membrane integrity of the cells is compromised. High-content fluorescence imaging was performed using the SpectraMax i3 platform with MiniMax Imaging Cytometer (Molecular Devices, Silicon Valley, CA, USA).

### 2.5. RNA Extraction and RNA m^6^A Determination

Total RNA was extracted from the lung tissue of mice and cultured cells using TRIzol reagent (Invitrogen, Carlsbad, CA, USA), according to the manufacturer’s instructions. After exposure to PM_2.5_, the lungs of mice and A549 cells were stored at −80 °C for further RNA extraction. Tissue lung RNA extraction requires sonication before adding TRIzol, while cell sedimentation can be completed directly with TRIzol. Then, add 1/5 TRIzol volume of chloroform. After standing on ice, the upper aqueous phase (including RNA) was retained after centrifugation. Then, an equal volume of isopropanol was added to the RNA aqueous phase. Wash off isopropanol with 75% ethanol (3 times). Finally, dissolve RNA with 20–40 μL DEPC water for storage. RNA was quantified by a Nano Drop 2000 spectrometer (Thermo Scientific, Waltham, MA, USA; Newburyport horse, MA, USA). Equal amounts of genomic RNA were analyzed for m^6^A level by using the EpiQuik m^6^A RNA Methylation Quantification Kit (Epigentek, Farmingdale, New York, NY, USA) according to the manufacturer’s instructions.

### 2.6. RT-PCR

The PrimeScript RT Master Mix (TaKaRa, Dalian, China) was used to synthesize cDNA. Real-time PCR validation was conducted using the Maxima^®^ SYBR^®^ Green qPCR Master Mix kit (CWBIOtech, Beijing, China) according to the manufacturer’s instructions, in an ABI Prism 7500 Sequence Detection System 288 (Applied Biosystems Inc., Waltham, MA, USA; Newburyport horse, MA, USA), with the following procedures: 95 °C for 10 min; 95 °C for 30 s, 60 °C for 1 min, and 72 °C for 1 min; 40 cycles. β-actin is used as a standardized internal control. The primers are shown in Table 2. Each sample was repeated in triplicate, and the fold change in gene expression was calculated according to the 2^−ΔΔCt^ method.

### 2.7. ELISA Analysis

The collected cells were thoroughly mixed with the lysate NP-40 (Beyotime, Shanghai, China) and centrifuged, and the supernatant lysate was taken for protein expression analysis. In this study, enzyme-linked immunosorbent assay (ELISA) was used to analyze the expression level of target protein in A549 cells. The ELISA kits were all purchased from Abcam (TGF-β1: ab100647; Smad2: ab260065; Smad3: ab264624; METTL3: ab163377; METTL14: ab163640; WTAP: ab100647; ALKBH5: ab162879; FTO: ab253759). All reagents, samples, and standards were prepared following the manufacturer’s instructions.

### 2.8. Use of TGF-β Inhibitors

TGF-β inhibitor fresolimumab (GC 1008) is a high-affinity, fully human monoclonal antibody that neutralizes the active forms of human TGF-β1, β2, and β3 [23]—adding it to the cell culture medium alone or together with PM_2.5_. The experimental group consisted of four groups: control group without any treatment (C); TGF-β inhibitor added group (G); PM_2.5_ added group (P); PM_2.5_ and TGF-β inhibitor simultaneously added group (PG).

### 2.9. Statistical Analysis

Statistical analysis was conducted using GraphPad Prism 8.0 software. All results are expressed as mean ± standard deviation. The statistical test uses the one-way analysis of variance. *p* < 0.05 was considered statistically significant.

## 3. Results

### 3.1. TGF-β1/Smad2/3 Levels in Lungs of Mice after Real-World PM_2.5_ Exposure in Chambers

**Figure 1:**
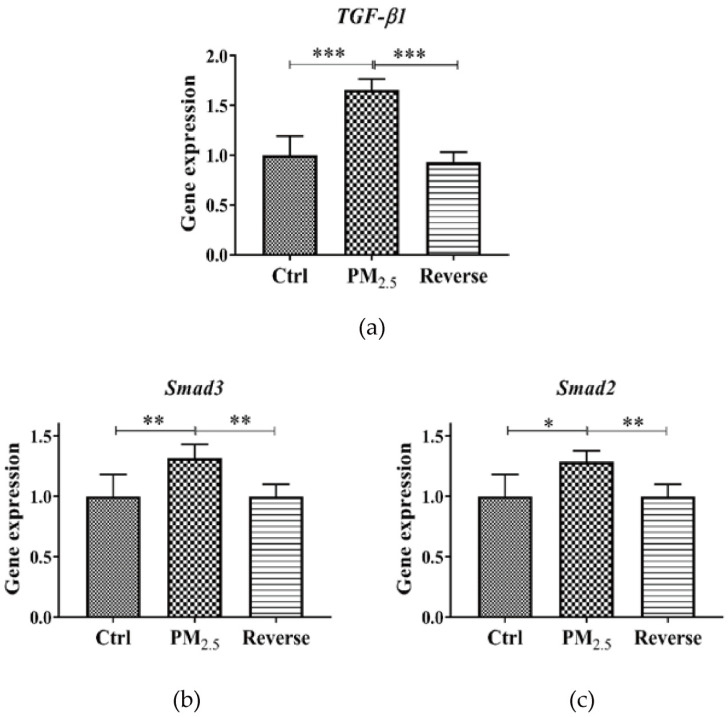
The expression of TGF-β1, Smad2/3 gene in lungs of mice after PM_2.5_ exposure. (**a**–**c**) PM_2.5_ exposure induced a significant up-regulation of TGF-β1 and Smad2/3 gene expression in mice lungs, and their levels were back to normal after air purification. * *p* < 0.05. ** *p* < 0.01. *** *p* < 0.001.

Our previous study showed that acute PM_2.5_ exposure induced a reversible change of RNA m^6^A levels and METTL3 and METTL14 in the lungs of mice [12]. Considering the change patterns of RNA m^6^A levels and METTL3 and METTL14, the expression of the TGF-β1 and Smad2/3 was also analyzed to explore whether TGF-β-Smad pathway is involved in PM_2.5_ exposure-induced m^6^A RNA methylation modification and to clarify its up and downstream relationships. We found that the expression of the TGF-β1 gene was significantly increased in the lung tissues of mice in the PM_2.5_ exposed group (*p* < 0.05) (Figure 1a). Meanwhile, the expression of the Smad2 and Smad3 gene were also elevated significantly compared to the control group (*p* < 0.05) (Figure 1b,c). All these alterations returned to normal after being transferred to purified air and continuing to live for 120 h.

### 3.2. Permeability of A549 Membrane after PM_2.5_ Exposure

**Figure 2:**
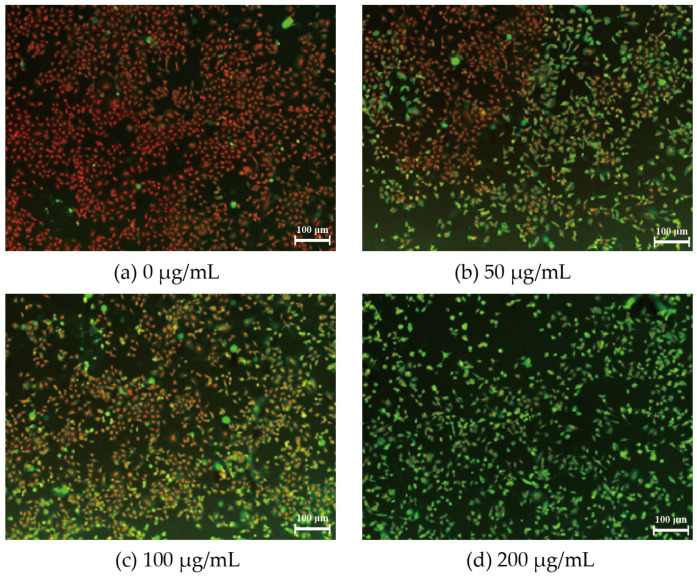
Fluorescence staining imaging results of A549 cells after PM_2.5_ exposure: (**a**) There is almost no green fluorescence, and the cells are impermeable without PM_2.5_ treatment; (**b**) After exposure to 50 μg/mL PM_2.5_, the green fluorescence signal is locally enhanced, and the cells are locally permeable; (**c**) After exposure to 100 μg/mL PM_2.5_, the green fluorescence signal is greatly enhanced, and the membrane was more permeable; (**d**) When the PM_2.5_ exposure concentration reaches 200 μg/mL, the cell membrane is completely permeabilized, and the cell survival rate is meager.

Membrane permeability is an effective indicator of cellular damage and can be visualized by the two-color nuclear staining assay with high-content fluorescence imaging. The green fluorescent signal represents damaged cells, while the red fluorescent signal indicates normal cells. In the present study, we found that the intensity of the green signal increased and the density of the cell decreased as we elevated the concentration of PM_2.5_ (Figure 2). When the PM_2.5_ concentration reached 200 μg/mL, the field of view was almost entirely filled with green signals, which meant that the cell membrane was completely permeated and cell survival was meager (Figure 2d). Therefore, the subsequent molecular biology analyses were performed at low to moderate concentrations of PM_2.5_ (0, 50, 100 μg/mL).

### 3.3. RNA m^6^A and TGF-β1 Levels in A549 after PM_2.5_ Exposure

**Figure 3:**
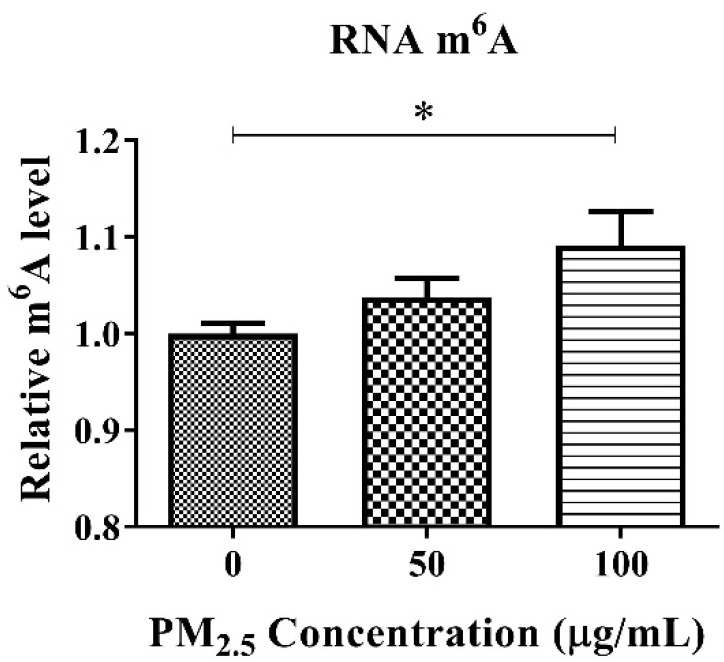
RNA m^6^A level in A549 cells after PM_2.5_ exposure. PM_2.5_ with a concentration of 100 μg/mL exposure induced a significant up-regulation of RNA m^6^A level in A549 cells. *: *p* < 0.05.

We determined the concentration of m^6^A RNA methylation in cultured A549 cells after PM_2.5_ treatment. The level of RNA m^6^A in A549 cells gradually increased with the increase in PM_2.5_ concentration gradients (0, 50, 100 μg/mL) (Figure 3). When the concentration of PM_2.5_ reached 100 μg/mL, the level of RNA m^6^A was significantly up-regulated (*p* < 0.05). Therefore, 100 μg/mL of PM_2.5_ was selected as the representative exposure concentration for the subsequent study.

**Figure 4:**
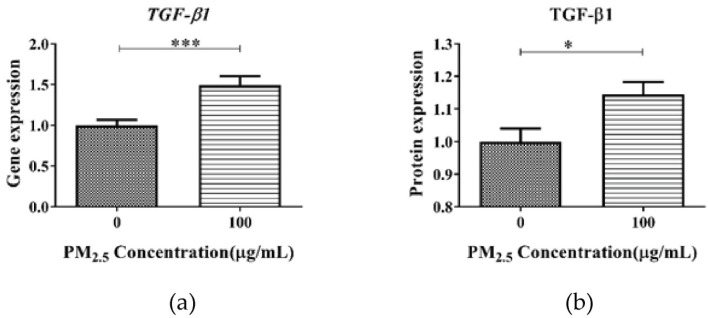
The expression of TGF-β1 gene and protein in A549 cells after PM_2.5_ exposure: (**a**) PM_2.5_ exposure induced a significant up-regulation of TGF-β1 gene expression in A549, (**b**) PM_2.5_ exposure induced a significant up-regulation of TGF-β1 protein expression in A549 cells. *: *p* < 0.05; ***: *p* < 0.001.

The expression level of the TGF-β1 gene and protein were detected. The results of qPCR and ELISA analysis showed that the expression of the TGF-β1 gene and protein in the A549 cell lysate treated with PM suspension was significantly higher than that in the absence of PM_2.5_ (*p* < 0.05) (Figure 4). These results indicate that TGF-β1 could mediate PM_2.5_-induced RNA m^6^A methylation in A549 cells.

### 3.4. Effect of TGF-β Inhibition on m^6^A RNA Methylation of A549 after PM_2.5_ Exposure

**Figure 5:**
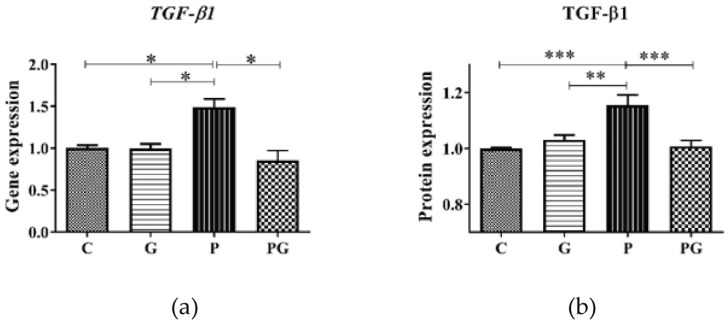
The expression of the TGF-β1 gene and protein of A549 cells of all groups. C: control group without treatment; G: TGF-β inhibitor added group; P: PM_2.5_ added group; PG: PM_2.5_ and TGF-β inhibitor simultaneously added group. (**a**) PM_2.5_ induced up-regulation of TGF-β1 gene expression of A549 was inhibited after TGF-β inhibitor treatment. (**b**) PM_2.5_ induced up-regulation of TGF-β1 protein expression of A549 was inhibited after TGF-β inhibitor treatment. *: *p* < 0.05; **: *p* < 0.01; ***: *p* < 0.001; no label means no statistically significant difference between groups (the same below).

**Figure 6:**
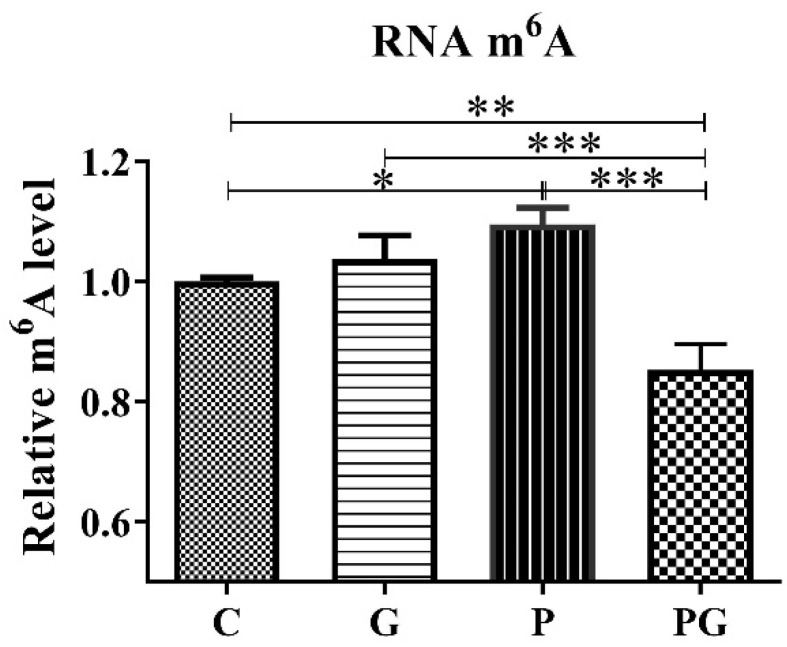
The RNA m^6^A levels of A549 cells of all groups. C: control group without treatment; G: TGF-β inhibitor added group; P: PM_2.5_ added group; PG: PM_2.5_ and TGF-β inhibitor simultaneously added group. PM_2.5_ induced up-regulation of RNA m^6^A level in A549 was reversed after TGF-β inhibitor treatment. *: *p* < 0.05; **: *p* < 0.01; ***: *p* < 0.001.

To further examine the role of TGF-β in PM_2.5_-induced m^6^A RNA methylation, the TGF-β inhibitor (GC 1008) was added to the cell culture medium alone or with PM_2.5_. First, each experimental group’s TGF-β1 gene and protein expression levels were detected. The results showed that the PM_2.5_-induced up-regulation of the TGF-β1 gene and protein expression was reversed after treatment with the TGF-β inhibitor (*p* < 0.05) (Figure 5), indicating that the inhibitor did suppress TGF-β1 expression. Further, the RNA m^6^A levels of each experimental group were analyzed. The results showed that the PM_2.5_-induced up-regulation of the RNA m^6^A level was reversed after treatment with TGF-β inhibitor (*p* < 0.05) (Figure 6), which directly indicated that TGF-β1 was involved in PM_2.5_-induced m^6^A RNA methylation.

### 3.5. Effect of TGF-β Inhibition on the Smad2/3 in A549 after PM_2.5_ Exposure

**Figure 7:**
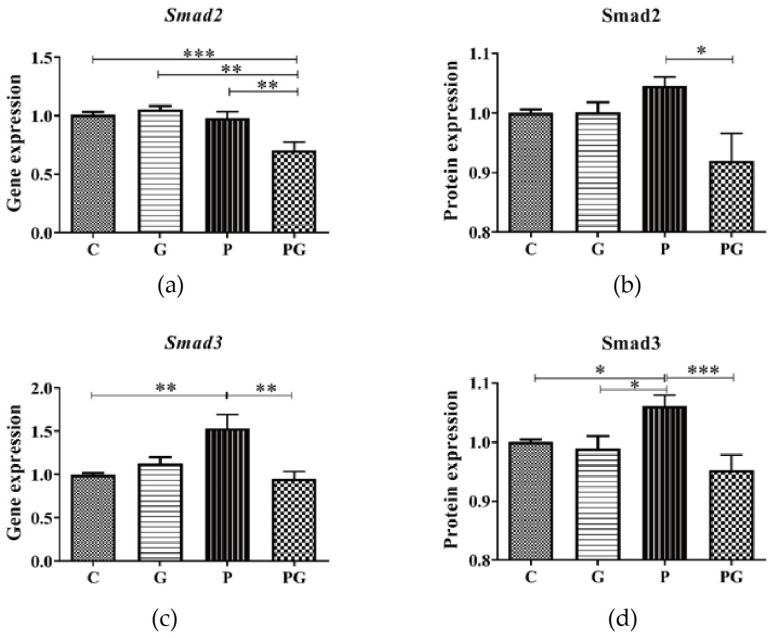
Smad2/3 level in A549 cells of all groups. C: control group without treatment; G: TGF-β inhibitor added group; P: PM_2.5_ added group; PG: PM_2.5_ and TGF-β inhibitor simultaneously added group. (**a**,**b**) Smad2 gene and protein expression did not change significantly after PM_2.5_ exposure. (**c**,**d**) PM_2.5_ induced up-regulation of Smad3 gene and reversed protein expression after TGF-β inhibitor treatment. *: *p* < 0.05; **: *p* < 0.01; ***: *p* < 0.001.

The results showed that there was no significant change in the Smad2 gene and protein expression in A549 after PM_2.5_ exposure. In contrast, Smad2 expression was significantly downregulated in the group treated with both PM_2.5_ and TGF-β inhibitors compared with the PM_2.5_-added group (Figure 7a,b). Furthermore, PM_2.5_ induced up-regulation of the Smad3 gene and protein expression in A549 was reversed after TGF-β inhibitor treatment (*p* < 0.05) (Figure 7c,d). These results indicated that TGF-β affected Smad3 expression in A549 after PM_2.5_ exposure.

### 3.6. Effect of TGF-β Inhibition on the RNA (de)Methyltransferase in A549 after PM_2.5_ Exposure

**Figure 8:**
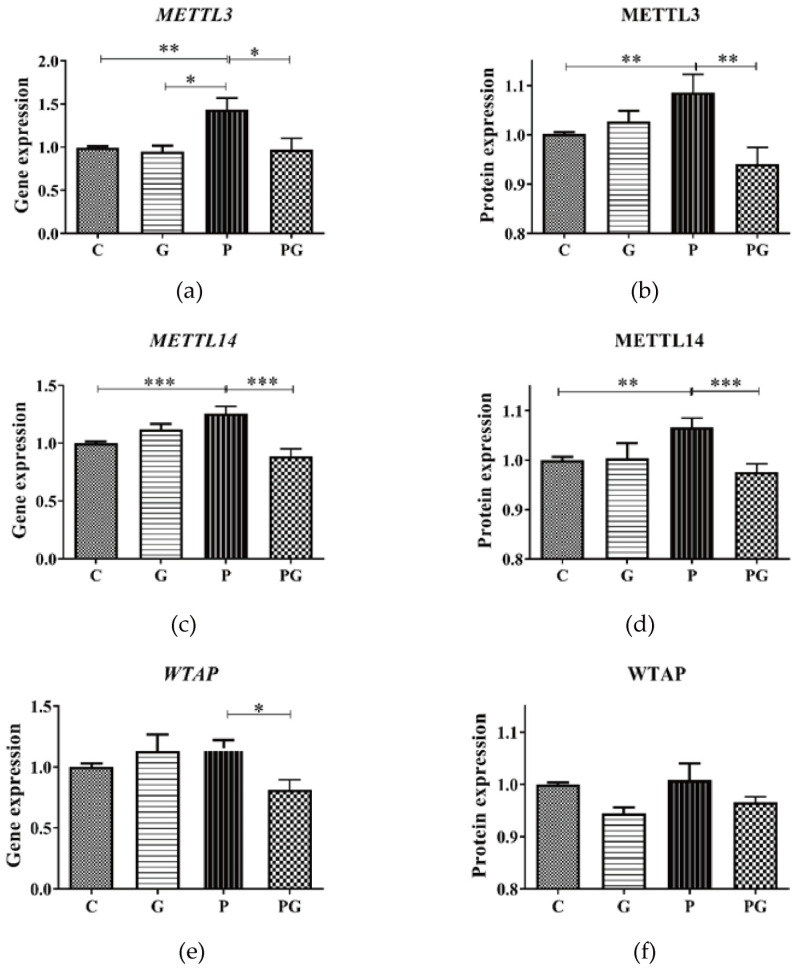
METTL3, METTL14, and WTAP level in A549 cells of all groups. C: control group without treatment; G: TGF-β inhibitor added group; P: PM_2.5_ added group; PG: PM_2.5_ and TGF-β inhibitor simultaneously added group. (**a**,**b**) PM_2.5_ induced up-regulation of METTL3 gene and protein expression in A549 was reversed after TGF-β inhibitor treatment. (**c**,**d**) PM_2.5_ induced up-regulation of METTL14 gene and protein expression in A549 was reversed after TGF-β inhibitor treatment. (**e**,**f**) WTAP gene and protein expression did not change significantly after PM_2.5_ exposure. *: *p* < 0.05; **: *p* < 0.01; ***: *p* < 0.001.

M^6^A RNA methylation is directly mediated by RNA methyltransferase and de-methyltransferase. Therefore, we analyzed the expression of RNA methyltransferase like-3 (METTL3), methyltransferase like-14 (METTL14), Wilms Tumor 1 associated protein (WTAP), and de-methyltransferase ALKB homolog 5 (ALKBH5), fat mass and obesity-associated protein (FTO). The results showed that PM_2.5_-induced up-regulated gene and protein expression of METTL3 and METTL14 in A549 were reversed after TGF-β inhibitor treatment (*p* < 0.05) (Figure 8a–d). WTAP gene and protein expression did not change significantly after PM_2.5_ exposure (Figure 8e,f).

**Figure 9:**
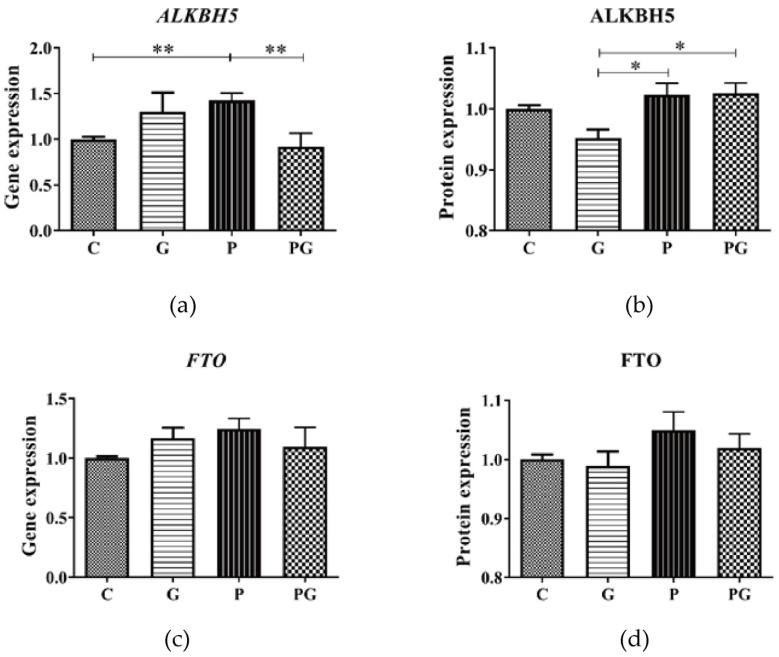
ALKBH5 and FTO level in A549 cells of all groups. C: control group without treatment; G: TGF-β inhibitor added group; P: PM_2.5_ added group; PG: PM_2.5_ and TGF-β inhibitor simultaneously added group. (**a**) PM_2.5_ induced up-regulation of ALKBH5 gene expression in A549 was reversed after TGF-β inhibitor treatment. (**b**) ALKBH5 protein expression did not change significantly after PM_2.5_ exposure. (**c**,**d**) FTO gene and protein expression did not change significantly after PM_2.5_ exposure. *: *p* < 0.05; **: *p* < 0.01.

In addition, at the gene level, PM_2.5_-induced up-regulation of ALKBH5 expression in A549 was reversed after TGF-β inhibitor treatment (Figure 9a). At the same time, no significant changes were observed at the protein level (Figure 9b). Similarly, FTO gene and protein expression did not change significantly after PM_2.5_ exposure (Figure 9c,d).

## 4. Discussion

Numerous studies have pointed out that epigenetic modification is involved in the adverse health effects after PM_2.5_ exposure [24]. Currently, m^6^A RNA methylation is a newly discovered and essential modification method in recent years, which regulates gene expression at the post-transcriptional level [25]. M^6^A modifications play a role in regulating gene expression, and their dysregulation could be linked to human diseases or cancer [10]. Previous studies have identified m^6^A as a significant substrate for FTO (de-RNA methyltransferase), suggesting that dysregulation of m^6^A RNA modification could be associated with disease [26]. The study found that METTL3 and METTL14 interact with chromatin and the transcriptional machinery [27], suggesting that disruption of this process could lead to the development of diseases, such as lung fibrosis and lung cancer metastasis [28]. Meanwhile, many researchers found PM_2.5_ linked to lung fibrosis and lung cancer [29,30]. These findings suggested the association between m^6^A methylation modifications and lung pathological changes caused by exposure to PM_2.5_.

However, at present, only a relatively small number of studies have begun to focus on the changes in m^6^A RNA methylation after PM_2.5_ exposure. The previous animal experiments of our team found that acute PM_2.5_ exposure can induce changes in RNA m^6^A, and this process is reversible [12]. Cayir et al. [31] found that exposure to PM at >62 μg/mL concentrations induced significant changes in m^6^A methylation in A549. In order to verify the effect of PM_2.5_ exposure on m^6^A RNA methylation and explore the possible regulatory mechanisms, this study was further investigated using an existing animal exposure model and a newly established cellular exposure model. Our study aims to elucidate the impact of PM_2.5_ exposure on RNA m^6^A methylation through TGF-β-related signaling pathways.

In the present study, we analyzed the level of global RNA m^6^A, and the expression levels of RNA (de)methyltransferase, TGF-β, and Smad2/3 after PM_2.5_ exposure in both animal and cellular studies. Our results showed that compared with the untreated group (control group), acute PM_2.5_ exposure resulted in increased global RNA m^6^A levels accompanied with increased expression of METTL3, METTL14, TGF-β, Smad3 in both lungs of mice and A549 cells. It is worth noting that PM_2.5_-induced changes in RNA m^6^A and the expression of METTL3, METTL14, TGF-β, and Smad3 could be reversed to normal when both TGF-β inhibitor and PM_2.5_ were added to the cell medium. These results suggested that exposure to PM_2.5_ leads to the regulation of m^6^A RNA methylation, and TGF-β could be an intermediate mediator. Xu Zihan et al. [32] found PM_2.5_ induced the uo-regulation of TGF-β1 of lungs in mouse. Phosphorylated Smad2/3 can interact with RNA methylation transferases METTL3, METTL14, and WTAP in the nucleus to regulate the m^6^A level of target mRNAs [19]. Based on this evidence, we hypothesized that the TGF-β/Smads pathway could be important to regulate RNA m^6^A modification related to PM_2.5_ exposure.

TGF-β superfamily is responsible for the initiation of the intracellular signaling pathways [33]. Cellular homeostasis, regulation of inflammation and immunity, extracellular matrix (ECM) synthesis, and many essential physiological processes depend on intact and appropriate TGF-β signaling [34]. Generally, the commonly accepted model of TGF-β signal transduction is that the TGF-β dimer binds to the TGF-β receptor, then sequentially transfers phosphate groups to Smad2 and Smad3 proteins, which are translocated to the target gene DNA sequence to activate or inhibit the expression of the target gene [35,36]. The core of this signal pathway is the Smad transcription factor.

To explore the possible regulatory role of TGF-β on m^6^A RNA methylation after PM_2.5_ exposure, we performed in vivo and in vitro experiments. The expression of TGF-β and key downstream factors of Smad2/3 in lungs of mice after PM_2.5_ exposure were analyzed. We found that the expression of TGF-β1 and Smad2/3 genes was significantly increased in the lungs of mice in the PM_2.5_ exposed group, while the expression of TGF-β1 and Smad2/3 genes returned to normal after they were transferred to clean air and continued to be fed for 120 h. Thus, at the animal level, the reversible changes between m^6^A RNA methylation and TGF-β and Smad2/3 gene expression after PM_2.5_ exposure are consistent, confirming a role for TGF-β in PM_2.5_-induced m^6^A RNA methylation. In order to clarify the role of TGF-β in the induction of m^6^A RNA methylation by PM_2.5_, we further experimented at the cellular level. After conducting cell mortality and permeability studies, we determined that 100 μg/mL of PM_2.5_ for 24 h was appropriate for the follow-up study. Consistent with our animal exposure study, PM_2.5_ exposure also significantly increased m^6^A RNA methylation, TGF-β gene and protein in A549. These results were similar to the results of most previous studies. Zheng et al. [37] found that 21 days of exposure to PM_2.5_, Balb/c mice showed increased TGF-β1 levels in the bronchoalveolar lavage fluid of the lung. Dysart et al. [38] found that exposure to PM_2.5_ resulted in increased activation of the TGF-β in ATII cells. The results of the more convincing TGF-β inhibitor study showed that when PM_2.5_ and TGF-β inhibitor were simultaneously added to the cell culture medium, compared with the control group, the expression of the TGF-β gene and protein did not increase significantly, and the RNA m^6^A level did not increase. These results fully demonstrated the indispensable role of TGF-β in PM_2.5_-induced m^6^A RNA methylation. Exposure to PM_2.5_ leads to upregulation of TGF-β expression, which regulates m^6^A RNA methylation via Smad2/3. Our study enhances the understanding of the relationship between TGF-β and epigenetic modifications following PM_2.5_ exposure.

Meanwhile, we found statistically significant upregulation of the Smad3 gene and protein expression in A549 after PM_2.5_ exposure, while the Smad2 gene and protein expression also tended to be upregulated. The study by Xu et al. [32] got similar results with us. They found chronic PM_2.5_ exposure in human bronchial epithelial cell line BEAS-2B cells led to the activation of the TGF-β1/Smad3 pathway. Singh et al. [39] also showed that the TGF-β/Smad3 pathway was activated in the diesel exhaust-exposed mice model. Notably, when PM_2.5_ and TGF-β inhibitors were added to the medium simultaneously, the expression of the Smad3 gene and protein was significantly upregulated compared to the control group, while Smad2 was not. This study suggests that exposure to PM_2.5_ increases TGF-β, which regulates Smad3 expression. After PM_2.5_ exposure, TGF-β induces m^6^A RNA methylation changes by regulating Smad3, which is independent of Smad2.

The m^6^A RNA methylation is directly mediated by RNA methyltransferase METTL3 and METTL14 and other cofactors such as WTAP RNA demethylation transferase as ALKBH5 and FTO can demethylate the modified m^6^A RNA [13]. To better explain the regulatory pathway of PM_2.5_-induced m^6^A RNA methylation, the effects of PM_2.5_ exposure on RNA methyltransferase and de-methyltransferase were also examined. The animal study showed that PM_2.5_ induced a significant increase in the expression of RNA methyltransferase METTL3 and METTL14 and that the change was reversible [12]. The A549 cell exposure experiment also showed the same results. Moreover, there was no significant change in ALKBH5 and FTO protein expression after PM_2.5_ exposure. These results suggest that PM_2.5_-induced m^6^A RNA methylation is mainly affected by METTL3 and METTL14. In other words, PM_2.5_ exposure is more likely to increase RNA m^6^A levels by adding methyl groups to RNA. Furthermore, when PM_2.5_ and TGF-β inhibitors were simultaneously added to the medium, compared with the control group, METTL3 and METTL14 did not increase significantly. This result suggests that TGF-β induced by PM_2.5_ regulates the expression of METTL3 and METTL14, thus m^6^A RNA methylation. Some research found that ALKBH5 knockout resulted in spermatogenesis defects due to elevated spermatocyte apoptosis [13]; disruption of the transcription process by METTL3 and METTL14 could lead to lung fibrosis and lung cancer metastasis [27,28]. M^6^A RNA methylation renders proliferation and progression of non-small cell lung cancer through regulating the TGF-β pathway [40]. This research indicates that alteration of m^6^A RNA methylation could play a crucial role in the occurrence and exacerbation of related diseases induced by PM_2.5_ exposure.

Two points about the limitations of this study are as follows: (1) The chemical composition of PM_2.5_ plays a crucial role in determining its impact on health outcomes. Differences in PM_2.5_ components are caused by variations in collection time and location across regions. Different components often have different effects. Therefore, whether the same research results can be obtained for PM_2.5_ collected from other places needs to be further studied. However, in our study, both the animal exposure study we reported before and this cellular exposure study showed the same results, i.e., PM_2.5_ exposure induced an increase in the level of m^6^A RNA methylation and METTL3 and METTL14. Thus, it is reasonable to acknowledge the accuracy of our results. (2) The previous published study suggested that phosphorylated Smad2/3 can interact with RNA methyltransferase complex to induce m^6^A RNA methylation [19]. In the present study, limited by the experimental conditions, we were temporarily unable to investigate the phosphorylation level of Smad2/3 and the interaction between Smad2/3 and RNA methyltransferase complex after PM_2.5_ exposure, which needs to be further explored. However, in any case, we have clarified that TGF-β plays an indispensable role in RNA methylation after PM_2.5_ exposure. Moreover, TGF-β regulation of Smad3, METTL3, and METTL14 could be one of the regulatory pathways of m^6^A RNA methylation after PM_2.5_ exposure.

## 5. Conclusions

Based on the present study, we confirmed that PM_2.5_ induced m^6^A RNA methylation, accompanied by increased TGF-β, Smad3, METTL3, and METTL14 expression. The TGF-β inhibitor experiments showed that TGF-β plays an indispensable role in m^6^A RNA methylation triggered by PM_2.5_ exposure. Therefore, we suggest that TGF-β regulates m^6^A RNA methylation by regulating the pathway of Smad3, METTL3, and METTL14 after PM_2.5_ exposure. Exposure to PM_2.5_ could cause changes in m^6^A RNA methylation levels through TGF-β, which could lead to various adverse health outcomes, such as lung fibrosis, lung cancer metastasis, and hindered organism development. Our study provides new insights into the pathogenesis and treatment of these diseases.

## Figures and Tables

**Table 1:** The average temperature and humidity during the sampling period (Beijing).

Time	Temperature (°C)	Humidity (%)
November 2019	1~9	20~46
December 2019	−3~4	54~86
January 2020	−9~2	39~53
February 2020	−6~5	53~86

**Table 2:** Sequence of primers of used for RT-PCR.

Gene	Sequence of Primer (5′→3′)	
	Forward	Reverse
	C57 BL/6J mice	
TGF-β1	AGCTGCGCTTGCAGAGATTA	AGCCCTGTATTCCGTCTCCT
Smad2	AGGATGATGGGGACGGGAAT	AGCCCGGTAAATCTACCCAGAA
Smad3	AGGAGAAGTGGTGCGAGAAG	CCATCCAGTGACCTGGGGAT
β-actin	CACTGTCGAGTCGCGTCC	TCATCCATGGCGAACTGGTG
	A549 cell	
TGF-β1	CAAGTGGACATCAACGGGTTC	TCCGTGGAGCTGAAGCAATAG
Smad2	ACCAAGCACTTGCTCTGAAA	ACGACCATCAAGAGACCTGG
Smad3	TAATTTATTGCCGCCGCTCG	ATCCAGGGACTCAAACGTGG
METTL3	GTGATCGTAGCTGAGGTTCGT	GGGTTGCACATTGTGTGGTC
METTL14	GGACCTTGGAAGAGTGTGTTT	GATCCCCATGAGGCAGTGTT
WTAP	GCTTCTGCCTGGAGAGGATT	GTGTACTTGCCCTCCAAAGC
ALKBH5	TGACTGTGCTCAGTGGATATG	TGACAGGCGATCTGAAGCAT
FTO	GAGCGCGAAGCTAAGAAACTG	TGGGGGTCAGATAAGGGAGC
β-actin	GCCGCCAGCTCACCAT	TCGTCGCCCACATAGGAATC

## Data Availability

The original data presented in the study are included in the article; further inquiries can be directed to the corresponding author.
